# Regulation of ALF Promoter Activity in *Xenopus* Oocytes

**DOI:** 10.1371/journal.pone.0006664

**Published:** 2009-08-17

**Authors:** Dan Li, Abbas Raza, Jeff DeJong

**Affiliations:** Department of Molecular and Cell Biology, University of Texas at Dallas, Richardson, Texas, United States of America; University of Munich and Center of Integrated Protein Science, Germany

## Abstract

**Background:**

In this report we evaluate the use of *Xenopus laevis* oocytes as a matched germ cell system for characterizing the organization and transcriptional activity of a germ cell-specific *X. laevis* promoter.

**Principal Findings:**

The promoter from the ALF transcription factor gene was cloned from *X. laevis* genomic DNA using a PCR-based genomic walking approach. The endogenous ALF gene was characterized by RACE and RT-PCR for transcription start site usage, and by sodium bisulfite sequencing to determine its methylation status in somatic and oocyte tissues. Homology between the *X. laevis* ALF promoter sequence and those from human, chimpanzee, macaque, mouse, rat, cow, pig, horse, dog, chicken and *X. tropicalis* was relatively low, making it difficult to use such comparisons to identify putative regulatory elements. However, microinjected promoter constructs were very active in oocytes and the minimal promoter could be narrowed by PCR-mediated deletion to a region as short as 63 base pairs. Additional experiments using a series of site-specific promoter mutants identified two cis-elements within the 63 base pair minimal promoter that were critical for activity. Both elements (A and B) were specifically recognized by proteins present in crude oocyte extracts based on oligonucleotide competition assays. The activity of promoter constructs in oocytes and in transfected somatic *Xenopus* XLK-WG kidney epithelial cells was quite different, indicating that the two cell types are not functionally equivalent and are not interchangeable as assay systems.

**Conclusions:**

Overall the results provide the first detailed characterization of the organization of a germ cell-specific *Xenopus* promoter and demonstrate the feasibility of using immature frog oocytes as an assay system for dissecting the biochemistry of germ cell gene regulation.

## Introduction

The factors and mechanisms that control transcriptional regulation in spermatocytes and oocytes of higher organisms have not been as well characterized as those in somatic cells [1]–[3]. This is due in part to the fact that germ cells cannot be propagated in cell culture and because cell-free extracts from complex tissues such as the testis are composed of contaminating subpopulations of germ cells and somatic cells. As a result, studies on the mechanisms of mammalian germ cell gene expression have tended to rely on somatic cell culture systems—where germ cell genes should normally be off—or on cell-free extracts derived from mixed somatic and germ cell populations from whole tissue sources.

Despite these issues, many regulatory factors have been proposed as regulators of germ cell gene expression. For a few of these, including CREMτ and alternative general transcription factors such as TRF2 and TAF105, gene knockouts have demonstrated specific effects on fertility [4]–[9]. In contrast, it has been more difficult to show that regulatory factors identified in transfected somatic cells or those identified by *in vitro* protein-DNA interactions have genuine physiological roles in regulating germ cell genes.

In this report we ask whether frog oocytes, used in the early studies of gene regulation to define core promoter elements such as the TATA box, would be useful for characterizing germ cell promoter architecture and regulation [10]–[12]. There are several advantages to this approach. First, immature oocytes (stages I-VI) from frogs are very large and promoter constructs can be tested for activity by direct injection into the oocytes themselves. Second, cell-free extracts can be made in sufficient quantities to allow biochemical studies. Third, the set of basal transcription factors responsible for promoter recognition in germ cells, including oocytes, consists of a physiologically appropriate set that includes TRF3, ALF, and several TAF variants [1], [13]–[17]. These factors are different from TFIID components used for promoter recognition and activation in somatic cells, making oocytes a natural environment for dissecting the mechanisms of germ cell gene regulation. Thus, the approach allows a germ cell-specific promoter to be matched with cells, in this case oocytes, where the endogenous gene would normally be on.

To evaluate the use of *Xenopus* oocytes as a germ cell transcription system we have used the ALF gene as the model. ALF is a paralog of the large (α/β) subunit of TFIIA [18], [19] and it plays a TFIIA-like role in stabilizing TBP (TFIID) to TATA elements within promoter DNA [20]. Characterization of the ALF promoter in mouse resulted in the identification of a number of candidate transcription factors, including possible somatic repressors. These factors included the zinc-finger proteins SP1, SP3, CTCF and the winged helix transcription factor RFX1 [21]. Studies on the ALF gene in *Xenopus* have shown that its expression is similar to that observed in mice and is restricted to spermatocytes and oocytes [22]–[25]. The germ cell-specific expression pattern suggests that the gene has retained the same functional role across these species and that the regulatory mechanisms that control expression are conserved.

In this report we have isolated the promoter from the *Xenopus* ALF gene using a PCR-based genome walking approach and characterized it with respect to initiation site by both RT-PCR and 5′-RACE analysis. Interestingly, the low level of sequence homology between ALF promoters identified in *X. laevis*, *X. tropicalis*, mouse, rat, human, chimpanzee, chicken, and others made it difficult to predict regulatory elements on the basis of sequence homology alone. Nevertheless, functional assays showed that the promoter is able to drive high level expression of a downstream reporter in microinjected oocytes. Fine-scale deletion and mutational analysis resulted in the definition of a very small (63 bp) promoter, making it one of the smallest active germ cell promoter sequences identified thus far. Additional promoter constructs were used to show that the promoter consists of two discrete elements, A and B, both of which were necessary for activity. Finally, mobility shift assays using oocyte-derived extracts revealed multiple complexes which interacted with the A and B elements. Overall, the results showed that frog oocytes provide an effective system to study core promoter architecture and regulatory factor interactions for germ cell specific genes.

## Materials and Methods

### Isolation of the X. laevis ALF promoter

The *X. laevis* ALF promoter was isolated with the Promoter-Finder system (Clontech). In brief, genomic DNA was prepared from liver and digested with blunt-end six-cutters HincII, PvuII, EcoRV, or StuI. After ligation to adaptor primers the resulting fragments were used as templates for PCR reactions with an adaptor-specific primer and a downstream gene specific primer (GSP1; 5′-GGGTTAGCCGAATGGGCCATGA-3′) derived from the *Xenopus* ALF cDNA [15]. After nested reamplification reactions with a second gene-specific primer (GSP2; 5′-GCCTAACCGGAAGTTGGAACCA-3′), PCR products were cloned into the pGEM T-easy vector (Promega) and sequenced. All of the products had perfect sequence identity to an overlapping section from the 5′-end of the ALF cDNA.

### Promoter constructs

A 1745 bp HincII fragment from the ALF promoter was cloned into the pGEM T-easy vector and recloned into the KpnI and BglII sites in the luciferase reporter vector pGL3-Basic (Promega). Using this construct (ALF1.7) as the parent, PCR-mediated deletions were prepared. For ALF1.7, ALF1.0, and ALF0.25 (ALF250), reactions contained primer LR1 (5′-GAGATCTGCCTAACCGGAAGTTGGAAC-3′) and either LF1 (5′-TGGTACCAATAGGG CTCGAGCGGCCGC-3′), LF2 (5′-TGGTACCTAGTATAGTTGTGCCATATC-3′), or LF3 (5′-TGGTACCTGAACATTCATCAGCAACTT-3′). The HSV-TK promoter in the control promoter vector pGL3TK was generated by PCR from the pRL-TK vector (Promega).

Additional deletion constructs were prepared using primers oriented so that they would extend in opposite directions around the ALF250 template, leaving gapped molecules that were then ligated to generate complete circles. For ALF205, ALF165, ALF125, ALF85 AND ALF45, the reactions contained a forward primer: N4796 (5′-AGAATTCGGTACCTATCGATAGAGAAATG-3′) and one of the following: Del205 (5′-TGAATTCGGCCTCTCAGCCCCTGACCATC-3′), Del165 (5′-TGAATTCGTAATAATCCCCTCCCCACATG-3′), Del125 (5′-TGAATTCGAAAGAT ACGTATAATATCGCG-3′), Del85 (5′-TGAATTCGACGCGCAAAAGTCACGTCAG C-3′), Del45 (5′-TGAATTCGTCAGACCGCAGGCGATTGAAC-3′) and LA63 (5′-TGAATTCGAACGCCCAACGCGTT-3′). Each primer also had an EcoRI restriction enzyme site “GAATTC” at the 5′-end. After PCR the vector-sized fragments were digested by EcoRI and self-ligated with T4 ligase (Promega) to generate circular plasmids. All constructs were sequenced to verify the deletion and to show that no mutations had been introduced into the luciferase open reading frame.

For the M1A series of constructs, mutations were introduced into the parent 85 bp wild type promoter (ALF85). For M1A, the first 6 bp from the 5′ end were converted to 5′-CTGCGC-3′; for M2A, 5 bp from 7 to 11 were mutated to 5′-GTTTT-3′; for M3A, 11 bp from 12 to 22 were mutated to 5′-*CCGCGG*CAGTGCAGTCG-3′ *(the first 6 bp of this sequence is a SacII restriction site); for M4A, 10 bp from 23 to 32 were mutated to 5′-CCGCGGCTTGCGGGTT-3′* and for M5A, the 8 bp from 33 to 40 were mutated to 5′-*CCGCGG*GCGCAATG-3′. *Deletion constructs D1A, D2A and D3A were generated by deleting 11 bp, 21 bp and 31 bp from the 3′ end of WTA respectively.*

The M5B series of constructs were based on the ALF85 construct to which an additional 11 base pairs of endogenous sequence had been added at the 3′ end (ALF85+). In addition, these constructs all contained an EcoR1 site within the mutated region. For M5B, 8 bp from 33 to 40 were mutated to (5′-*GAATTC*TG-3′); for M6B, 12 bp from 41 to 52 were mutated to “TGGTT *GAATTC*T”; for M7B, 12 bp from 53 to 64 were mutated to 5′-TGT*GAATTC*GTG-3′; for M8B, 12 bp from 65 to 76 were mutated to (5′-TTG*GAATTC*GGG-3′); *for M9B, 9 bp from 77 to 85 were mutated to (5′-GTGAATTCG-3′);* for M10B, 11 bp from 86 to 96 were mutated to (5′-*GAATTC*TGTTG-3′). *The AM1-AM10 and BM1-BM6 series of mutant constructs were prepared using specific PCR primers, and the exact sequences are shown in the relevant figure.*

Mutant constructs with rearranged or respaced A and B elements were made as follows. For ALF63+5 the insert was 5′-CTCGA-3′, for ALF63+10 the insert was (5′-CTCGAGTCGC-3′, and for ALF63+30, the insert was 5′-GACGTCGCATACTCGAGGCCGCCATTACCT-3′. For ALF63-5 the deleted sequence was 5′-GCGAT-3′; for ALF63-10 the deletion was 5′-GCGATTGAAC-3′, and for ALF63-15 the deletion was 5′- GCGATTGAACGTGGT-3′. For ALF63+256, an insert of 256 bp sequence from a *X .laevis* actin cDNA was cloned in between the A and B elements in ALF63. Additional mutants MA, MB, and MAB mutants were made based in the ALF63+256 construct. In MA, element A was mutated to 5′-ACGGCATGACTC-3′. In MB, element B was mutated to 5′-TTGGGAGCC-3′. In MAB, both elements were mutated.

### Bisulfite methylation analysis

Genomic DNA was isolated from *Xenopus laevis* oocytes and liver using the Wizard SV Genomic DNA Purification System (Promega) and processed using the EpiTect Bisulfite Kit (Qiagen). In brief, DNAs were subject to repeated denaturing (99°C for 5 min) with several incubation steps (60°C for 25 min, 85 min, and 175 min). Reactions were cleaned up with EpiTect spin columns (Qiagen) and purified DNA was used as a template for PCR reactions with modified ALF promoter specific primer F (5′-ATGTGTTTTTTGAATATTTATTAGTAAT-3′) and primer R (5′-ATCTCCCATAACTACTTTAATTCCTTAAAC-3′). Final products of 275 bp were cloned into the pGEM T-easy vector (Promega) and sequenced.

### Preparation of oocytes and kidney cells

Oocyte-positive *Xenopus laevis* females (NASCO) were anesthetized with ethyl 3-aminobenzoate methanesulfonate and oocytes were removed surgically. Isolated oocytes were treated with 0.2% collagenase (Invitrogen) in OR2 medium for 3 hours at RT. Oocytes were then washed with OR2 media and incubated with penicillin-streptomycin solution (Sigma) at 20°C overnight. Stage V and VI oocytes were selected under an Olympus SZ-40 stereomicroscope. *Xenopus laevis* kidney epithelial cells XLK-WG (ATCC) were cultured at 32°C in complete growth medium composed of 60% RPMI 1640 and 20% FBS.

### Oocyte microinjections, cell transfections, and luciferase assays

For microinjections, ∼2 ng DNA was injected into 30 stage V/VI oocytes using a Nanoject II injector (Drummond) and TIP10XV119 needles (World Precision). After 24 hour incubation in OR2 media, healthy oocytes were selected and centrifuged for 10 min at 14,000 rpm at 4°C and the aqueous supernatant was collected. Extracts were diluted 100-fold and luciferase activity was measured in a Turner TD-20 luminometer with the Luciferase Assay System (Promega). Reporter activity was normalized to measured protein concentrations and each experiment was performed a minimum of three times and in most cases four to six times using oocyte preparations from different frogs. This repetition was important to control for interindividual differences in oocyte quality and microinjection efficiency.

XLK-WG kidney cells were grown to 50–80% confluence on 6-well plates and transfected with 1 µg of DNA using 3 µl of the FuGENE 6 reagent (Roche Applied Science). The pGL3-basic vector served as the negative control. Whole cell extracts were prepared after 24 h and luciferase activity was assayed.

### Analysis of RNA by Northern blot

Northern blotting of RNA from microinjected promoter constructs was performed as follows. RNA prepared from injected and uninjected oocytes was isolated using the Trizol reagent (Invitrogen). RNAs (12 µg) were loaded onto a formaldehyde-containing gel and transferred to a Zeta-Probe Blotting Membrane (Bio-Rad). The PCR-generated hybridization probe spanned nucleotides 128–771 of the luciferase reporter and was labeled with [α-^32^P] dCTP using Ready-To-Go DNA labeling beads (Amersham). A 260 bp PCR fragment from the *X. laevis* 5S RNA gene was used as an RNA normalization control.

### RT-PCR and 5′-RACE

Total oocyte RNA (5 µg) was used as template to prepare first strand cDNA with SuperScript II reverse transcriptase (Invitrogen) and oligo dT primers. First strand cDNAs were then used as templates in PCR experiments. Transcription start site usage for the endogenous ALF gene involved primers S0 (5′-GATCATGGCCCATTCGGCTAACC-3′), S1 (5′-TGAACATTCATCAGCAACTTGG-3′), S2 (5′-CCCCTGACCATCAATAAAACAC-3′), S3 (5′-TGCGCAGACATGAGCCAGCGGA-3′), S4 (5′-GCAGCAGCGCGACGCGCAAAAG-3′), S5 (5′-GTCAGACCGCAGGCGATTGAAC-3′), and S6 (5′-TGGTTCCAACTTCCGGTTAGGC-3′). Each primer was used together with a common downstream primer GSP1 (5′-CCTGTTGAGGTGTGAAGGGAGT-3′). 5′-RACE reactions were performed with the 5′-RACE System (Invitrogen) using primers GSP2 (5′-TTGTCCTCCAACTGCCTCAGA-3′) and nested primer GSP3 (5′-CCAAGACAACAGCACCCCACAA-3′).

### Electrophoretic mobility shift analysis

Oligonucleotide probes from the *X. laevis* ALF promoter used in bandshift assays were prepared by T4-kinase labeling with [γ-^32^P] ATP. Oocyte extracts were prepared from collagenase treated oocytes centrifuged twice at 14000 rpm at 4°C for 15 min in HB buffer (50 mM Tris-Cl, pH 7.5, 10% glycerol, 5 mM Mg-Acetate, 0.2 mM EDTA, 0.5 mM DTT, and 0.1 mM PMSF) in the presence of 1% protease inhibitor (Sigma).

Bandshift reactions typically contained 25 µg whole cell extract along with the DNA probe in EMSA reaction buffer (10 mM HEPES (PH 7.9), 2% PEG-8000, 5 mM dithiothreitol, 0.2%EDTA, 5 mM ammonium sulfate and 8% glycerol, 100 mM KCl, 5 mM MgCl_2_ and 2 µg poly dI-dC). Reactions were incubated at RT for 30 min and separated on 5% native acrylamide gels.

## Results

### Identification of the Xenopus laevis ALF promoter

**Figure 1:**
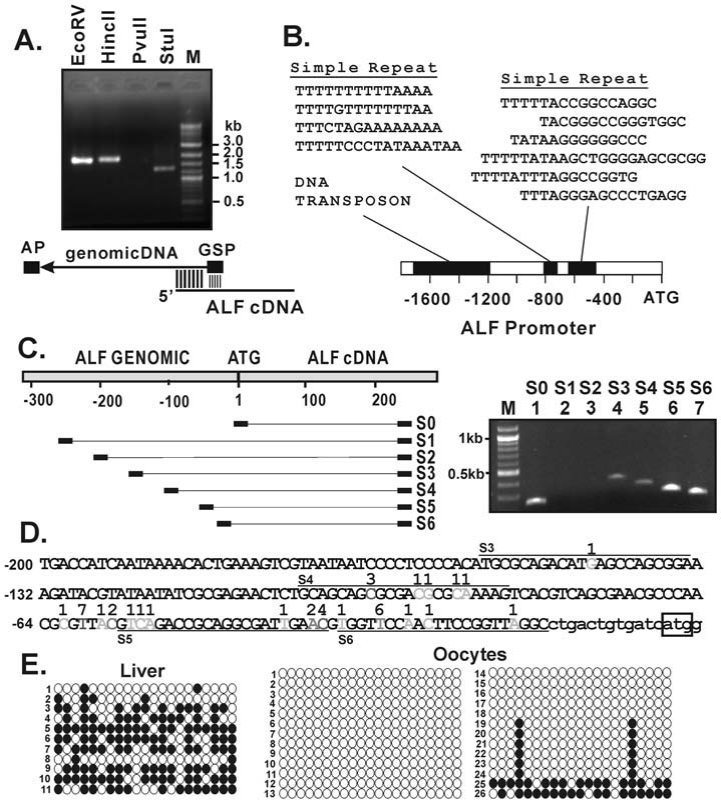
Isolation and transcription start site mapping of the *Xenopus* ALF promoter. (A) PCR reactions were performed with *X. laevis* genomic DNA that had been digested with EcoRV, HincII, PvuII, and StuI. The gene specific primer (GSP) was located 70 base pairs downstream of the 5′ end of ALF mRNA. AP is the adaptor primer. (B) The 1.7 kb HincII ALF promoter fragment contains a DNA transposon and two other repeats, examples of which are aligned in the figure. (C) To map the start site, RT-PCR reactions were performed with oocyte RNA using primers located at various locations throughout the ALF promoter region (S0-S6). The results show strong bands with primers S5 and S6 (lanes 6, 7), weaker bands with primers S3 and S4 (lanes 4, 5), and no bands with primers S1 and S2 (lanes 2, 3). (D) Sequence analysis of nearly 40 RACE clones shows the distribution of start sites throughout the promoter region. The number of hits observed at each position is indicated. Locations of the ATG and primers S3-S6 are indicated. (E) Sodium bisulfite methylation analysis of the ALF promoter shows a high degree of methylation (filled circles) in liver tissue where the gene is normally off, and little to no methylation (open circles) in oocytes where the ALF gene is normally on. Filled cirlces represent methylation while open circles represent no methylation.

Since the genome sequence of *X. laevis* is not available, the first step in this project was to isolate the promoter of the ALF gene from purified genomic DNA. This was accomplished by a genome walking approach that involved an upstream-directed gene specific ALF primer located within the 5′-UTR (Figure 1A). The largest product (1745 bp) was generated with HincII digested DNA. This product, as well those derived from DNA digested with other enzymes, showed an identical match to 70 base pairs in the 5′-UTR of the ALF cDNA, confirming that the genomic sequences are directly upstream of the ALF mRNA.

Computer analysis of the 1745 bp HincII sequence revealed several unique sequence features, including a 587 bp long DNA transposon (position −1739 to −1203) (Figure 1B). In addition, two short repetitive motifs were identified by visual inspection. One of these had an approximately 15 bp long “TA” rich fragment repeated at least four times, while the other was composed of six repetitions of a “TA-GC” rich element with length of about 15–20 bp (Figure 1B).

### Mapping the ALF initiation site

We determined the transcription start sites of the endogenous ALF gene using two different approaches. One approach involved a series of upstream primers located at various positions within the putative promoter region. These primers were used in combination with a common downstream primer to generate RT-PCR products from oocyte RNA (Figure 1C). The downstream primer was positioned so that amplification would occur only from spliced mRNAs and not from contaminating genomic DNA. Control reactions included a primer (S0) that was located a known distance (256 bp) upstream of the common primer. Results with primers S3, S4, S5, and S6 all generated PCR products corresponding in size to primer location, indicating that ALF mRNAs spanning these regions did exist (Figure 1C lanes 4, 5, 6, 7). Among the products, those derived from primers S5 and S6 (lanes 6, 7) were of greater intensity than those derived from primers S3 and S4 (lanes 4, 5). The results place the most upstream initiation site somewhere between the S2 and S3 primers, and place a stronger downstream site between the S4 and S5 primers. The failure of S1 and S2 to generate a signal (lanes 2 and 3) defines an upper limit beyond which transcription does not initiate. Overall, the results show that transcription can begin as far as 160 bp upstream of the ATG codon, but that the strongest signals occur approximately 50 bp upstream.

A second approach to map the ALF gene start site involved 5′-RACE analysis. In these experiments a primer located approximately 200 bp downstream of the ATG start codon was used for first strand cDNA synthesis reactions followed by reamplification with an adaptor primer and a nested gene-specific primer. The resulting PCR products were cloned and sequenced to determine their endpoints. The results, summarized in Figure 1D, reveal multiple start sites with frequencies ranging from one and seven. Most sites (∼80%) mapped between 16 to 66 nucleotides upstream of the ATG codon, consistent with the strong RT-PCR signals observed with primers S5 and S6 in Figure 1C. The most distal RACE product (observed once at position −146) was at a position consistent with signals observed with primer S3. Sequencing showed that all the RACE clones matched genomic DNA and there was no evidence for an upstream exon. Collectively, the promoter isolation and start site mapping experiments led us to conclude that we had correctly isolated the *X. laevis* ALF promoter region, and showed that the gene possessed multiple transcription start sites.

Many germ cell promoters are hypomethylated when active in germ cells and hypermethylated when silenced in somatic cells [24], [26], [27]. To test whether this was also true for the *Xenopus* ALF gene we performed bisulfite methylation analysis of genomic DNA isolated from liver and oocyte tissue. The results with 11 liver-derived clones showed an average methylation in the ALF promoter region of 55%, whereas oocyte-derived clones showed an average methylation of 8% (Figure 1E). Except for clones numbered 25 and 26, the remainder of the samples were either unmethylated or were methylated at only two positions. Since the DNA used in this experiment was derived only from oocytes, together with any contaminating follicle cells, the results suggest an association between a demethylated promoter state and activity.

### Alignment of ALF promoters from different species

We next asked whether homology between the *X. laevis* ALF promoter and corresponding ALF promoters from other organisms would help identify functional sequence elements. To address this question, the ALF gene from *X. laevis* was compared to those from human, chimpanzee, macaque, mouse, rat, horse, pig, cow, dog, chicken, and the frog *Xenopus tropicalis*. The sequences were identified by BLAST search and included ∼140 bp upstream of the first exon.

**Figure 2:**
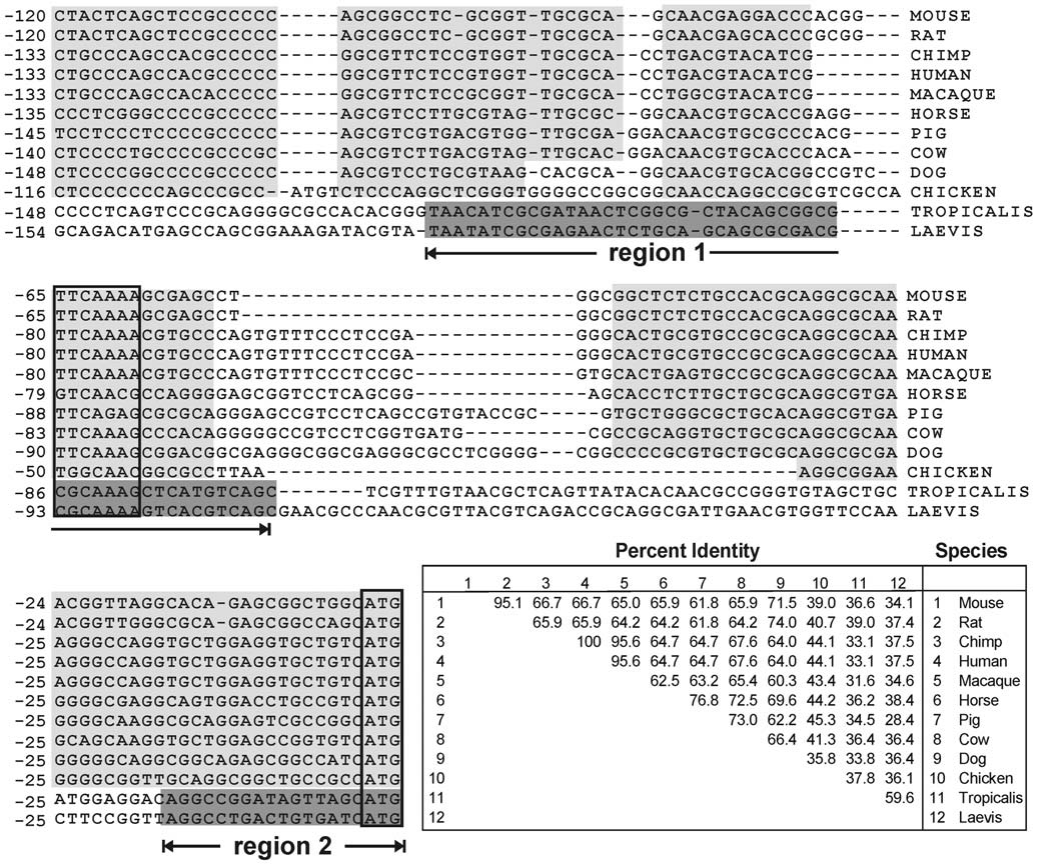
Alignment of ALF promoter sequences from different species. ALF promoters from twelve different species (mouse, rat, chimp, human, macaque, horse, pig, cow, dog, chicken, *X. tropicalis*, and *X. laevis*) were aligned. Conserved regions (shaded) could be identified among the first ten of these organisms. The two frog-derived sequences were only weakly similar and were shaded separately. The table shows pairwise identity scores.

Comparisons among these sequences showed that they can be grouped into two main categories (Figure 2). The first category include ten sequences including nine from mammals and one from chicken. A separate second category included the two frog sequences, *X. laevis* and *X. tropicalis*. Despite the fact that the sequences in both categories are germ cell specific, the sequences were quite divergent and did not enable the identification of putative functional elements based on homology alone. The highest pairwise alignment scores occurred between highly related organisms such as human and chimpanzee (100%), human and macaque (95.6%), and mouse and rat (95.1%). Interestingly, alignment of the two frog sequences, *X. laevis* and *X. tropicalis*, showed a comparatively weak similarity of 59.6%, presumably reflecting evolutionary distance and pseudotetraploidization in *X. laevis* [28], [29]. However, the two frog sequences did display two homologous subregions. One of these (region 1 in Figure 2) was 71.9% similar over 32 nt, while another (region 2), just upstream of the ATG codon, was 73.7% similar over 19 nt.

### Identification of a minimal ALF promoter of 63 bp

**Figure 3:**
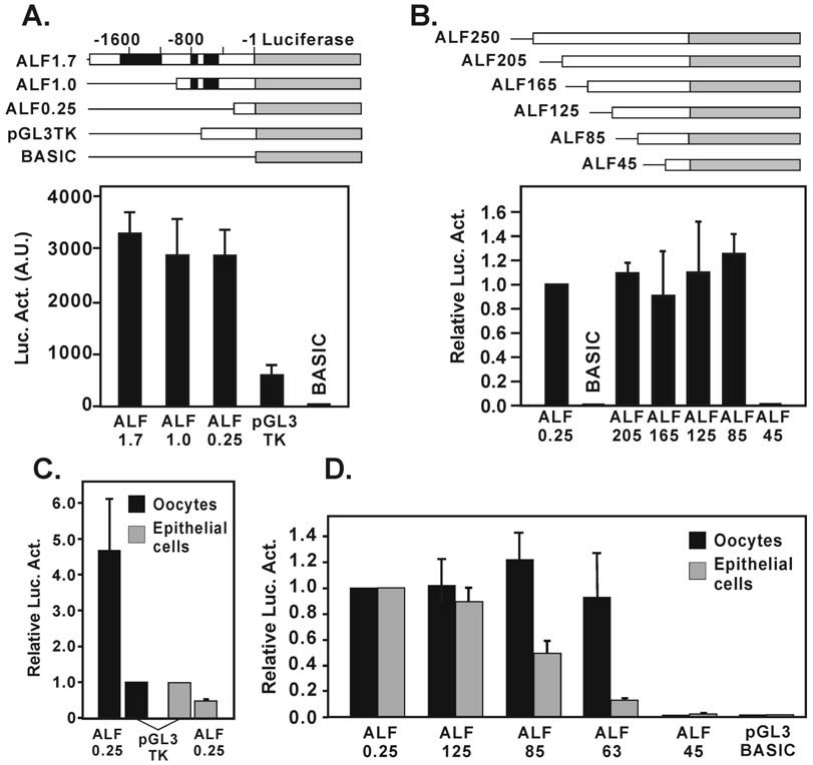
Deletion analysis of ALF promoter constructs reveal a very short active region. (A) Three promoter constructs, ALF1.7, ALF1.0, and ALF0.25 were linked to a luciferase reporter, microinjected into oocytes, and assayed for activity relative to controls pGL3TK (thymidine kinase) and an empty vector (pGL3BASIC). (B) Microinjection experiments with shorter deletions constructs prepared from the ALF0.25 parent (ALF250, ALF205, ALF165, ALF125, ALF85, and ALF45) showed that an 85 base pair construct retained full activity. (C) The relative activity of the ALF0.25 construct was compared in oocytes and *Xenopus* XLK-WG kidney epithelial cells in comparison to a pGL3-TK reference. (D) The relative activities of differently sized ALF constructs differ in oocytes and XLK-WG epithelial cells. The ALF0.25 construct served as the normalization control.

Microinjection experiments initially involved three constructs, a full-length version (ALF1.7) and two 5′ deletions (ALF1.0 and ALF0.25), all of which were designed to drive a luciferase reporter in the pGL3-Basic vector (Figure 3A). Construct ALF1.0 removed the DNA transposon while construct ALF0.25 removed both the transposon and the simple sequence repeats. A positive control construct placed the luciferase reporter under the control of the herpes simplex virus thymidine kinase (HSV-TK) promoter, while the empty vector served as a promoterless negative control (pGL3 Basic). Constructs were injected into stage V/VI *Xenopus* oocytes and assayed for luciferase after 24 hours of incubation. The results showed that the ALF constructs were all active, and that even the shortest ALF0.25 construct retained as much activity as ALF1.7 (Figure 3A). The low activity of the positive control showed that it was not as active here as would typically be expected in somatic cells.

An additional series of promoter deletions, ALF250, ALF205, ALF165, ALF125, ALF85, and ALF45, were also constructed. All of these were active except for ALF45 in which sequences between 85 and 45 had been removed (Figure 3B). This observation showed that the region required for full activity was 85 bp or less.

Although ALF and other germ cell genes are normally expressed only in germ cells, they typically will show some activity when introduced into somatic cells. Here we compared the relative activity of the ALF0.25 construct in both oocytes and in XLK-WG kidney epithelial cells (Figure 3C). The results showed that the relative activity of the ALF construct was about 4-fold higher in oocytes compared to a pGL3-TK control, whereas in somatic cells the activity was about 2-fold lower compared to that same control. We also tested the relative activity of various ALF promoter constructs after transfection into XLK-WG cells and after microinjection into oocytes. The results, normalized to the activity of ALF250, showed that the deletion constructs retained full activity in oocytes until sequences between 63 and 45 were removed (Figure 3D). In contrast, the activity of these same constructs in kidney cells showed a progressive length-dependent decline in activity. Although the basis for this decline is not known, the differences highlight the fact that somatic cells, although capable of supporting some level of transcription, do not exhibit the same regulatory profile as seen in oocytes. The use of the ALF63 construct in these experiments further refines the minimal ALF promoter from 85 base pairs to a region of not more than 63 nucleotides. The very small size of the promoter is consistent with earlier observations on mammalian germ cell promoters [22].

### Identification of the core promoter elements

**Figure 4:**
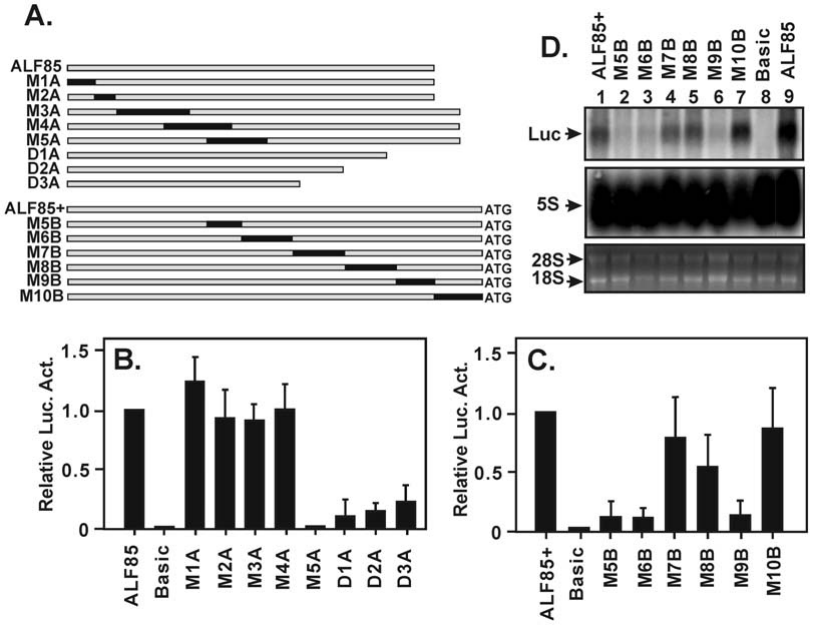
Identification of two core promoter elements. (A) A series of mutations and deletions were introduced into the ALF promoter and tested for their effect on activity. Constructs contained nucleotide substitutions (shown in black) or 3′-end deletions (D1A, D2A, and D3A) compared to the wild type controls ALF85 and ALF85+. (B) A set of ALF85 derived constructs injected and tested for activity showed diminished activity with M5A and all the 3′-deletions. (C) A set of ALF85+ derived constructs showed two regions with diminished activity, defined by constructs M5B/M6B and M8B/M9B. (D) Northern blot analysis of wild type and mutant constructs. The top panel shows luciferase RNA levels, the middle panel shows a control 5S rRNA gene hybridization, and the bottom panel shows ethidium bromide-stained 28S and 18S RNA.

To determine the location of elements within the ALF promoter that are critical for activity in oocytes, several additional series of mutation/deletion constructs were made. One such series involved base substitutions between −85 and −45 in the ALF85 parent, along with three deletion mutants D1A, D2A, and D3A (Figure 4A). Mutant M5A and all three deletion mutants showed a dramatic loss of luciferase activity (Figure 4B). Surprisingly, alteration of the CGCAAA sequence, which appeared to be similar to a conserved TTCAAA motif in the mammalian sequences (Figure 2), had no effect on transcription.

Because the D1A deletion mutant was close to the junction between the promoter and reporter, and because that junction included a potential CA initiation site, we wanted to exclude the possibility that construct design had resulted in new sites of transcription initiation. We therefore created a second series of mutants based on a parent construct ALF85+ that contained 11 additional nucleotides extending downstream to the ATG translation initiation codon (Figure 4A). Microinjection assays of these mutants revealed two regions that were critical for full activity (Figure 4C). One of these was defined by mutant constructs M5B and M6B and which were overlapping with M5A. This was termed the A element. The second region was defined by mutant constructs M8B, M9B, and deletion construct D1A. This was termed the B element. The similarity in the results obtained using two different series of mutant constructs strongly suggested that A and B were bona fide regulatory elements.

Luciferase assays measure the amount of reporter protein available at the time of assay and reflect the combined effects of regulation at the transcriptional and translational levels. Because the A and B elements were close to the ATG codon and potentially within the 5′-UTR of the transcribed RNA itself, we asked if their effects might be translational rather than transcriptional. To address this issue Northern blots were performed with RNA prepared from microinjected oocytes and a luciferase-specific hybridization probe (Figure 4D). The results showed that different constructs generated different steady-state levels of reporter RNA, and that the amount of RNA correlated well with the amount of luciferase activity. The results suggest that the mutations affect transcription and therefore define the location of promoter regulatory elements.

### Fine structure mapping of core promoter elements A and B

**Figure 5:**
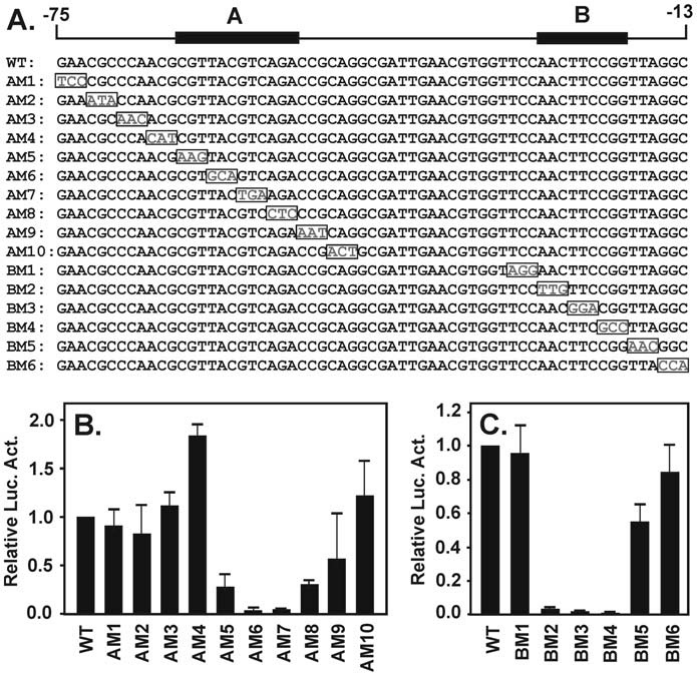
Fine structure mapping of the core promoter elements A and B. (A) A series of three nucleotide substitutions were made in and around the functional elements defined in the previous figure. (B) and (C) Constructs were injected, assayed for luciferase activity, and normalized to a WT (ALF63) control. The results define an upstream A element of about 12 nucleotides (CGTTACGTCAGA) and a downstream B element of about 9 nucleotides (AACTTCCGG).

To further narrow the location and sequence of the A and B elements we prepared a series of triplet nucleotide substitutions that covered the A (AM1 to AM10) and B (BM1 to BM6) elements (Figure 5A). The parent for these constructs was ALF63, the shortest region which retained full activity. Microinjection results for the A-series show diminished activity for the AM5, AM6 AM7, and AM8 constructs and normal activity in the others (Figure 5B). Results with the B-series of mutants showed a loss of activity for BM2, BM3 and BM4, but normal activity for BM1 and BM5 (Figure 5C). Overall, the data show that the promoter contains two distinct functional elements, A (5′-GCGTTACGTCAGA-3′) and B (5′-AACTTCCGG-3′).

### Protein complex formation on the ALF promoter

**Figure 6:**
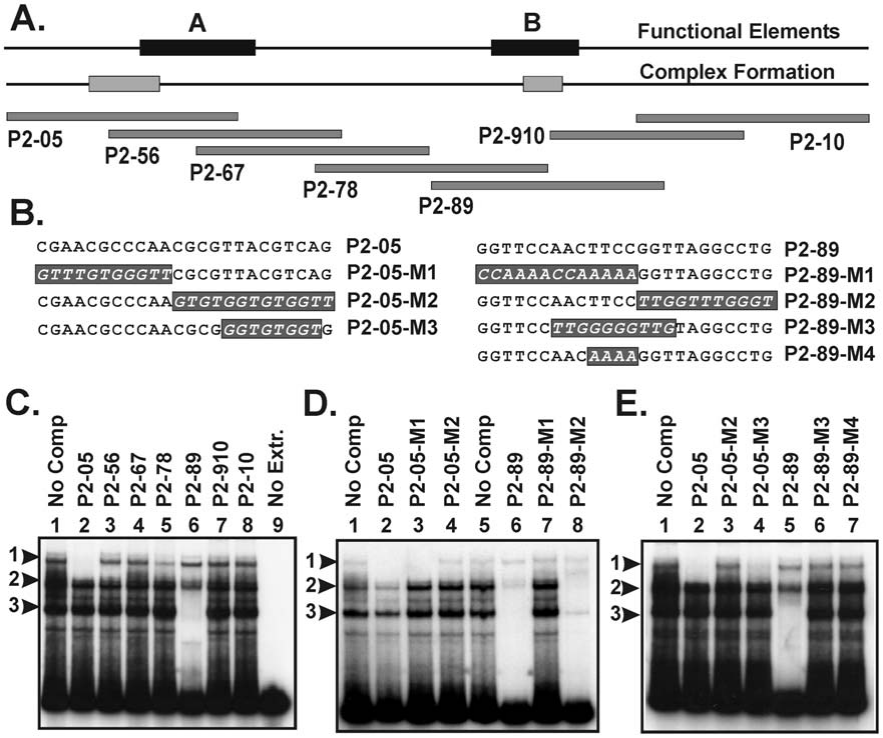
Identification of oocyte proteins that interact with the A and B elements. (A) A 96 bp fragment from the ALF85+ promoter was labeled and used as the probe in EMSA assays with oocyte extracts. A summary of the position of the A and B elements and the factor binding sites are shown in the two top lines. Beneath this is shown the relative locations of a series of overlapping oligonocleotide competitors. (B) An additional set of oligonucleotide competitors that contained specific mutations were also used as competitors in the binding assays. (C) Bandshift analysis shows the ALF promoter forms several protein-DNA complexes using oocyte-derived cell-free extracts. The main complexes are indicated by the labels 1, 2, and 3. The P2-05 competitor selectively abolishes complex 1 (lane 2), while the P2-89 competitor selectively abolishes complex 3 and to a lesser extent complex 2 (lane 6). (D) Additional competition assays show that P2-05-M1 but not P2-05-M2 is able to compete for complex 3 (compare lanes 3 and 4). Similarly, the P2-89-M2 competitor but not P2-89-M1 is able to compete for binding of complexes 2 and 3 (compare lanes 7 and 8). (E) Competition with mutant oligos P2-05-M3, P2-89-M3, and P2-89-M4 further refines the binding site to the positions noted in the ‘Complex Formation’ line in (A).

We next asked if DNA binding proteins in oocyte extracts could recognize the ALF promoter and whether the sites of factor interactions coincided with the locations of the A and B elements. To address this point, mobility shift assays were performed using an ALF85 promoter fragment as a probe, together with whole cell extracts from stage V/VI oocytes (Figure 6A, 6B). In the absence of any competitor three main complexes were observed (Figure 6C, lane 1). Competition for complex 1 occurred when oligo P2-05 was added (lane 2), and partial to complete competition occurred for complexes 2 and 3 when oligo P2-89 as added (lane 6). The use of an additional set of mutant competitors based on P2-05 and P2-89 were used to narrow down the likely region of binding (Figure 6D, 6E). As summarized in Figure 6A, complex 1 bound near a palindromic sequence AACGCGTT that partially overlapped with functional promoter element A. Partial competition for complex 1 was also observed with competitor P2-78 (Figure 6C, lane 5), perhaps because it contained an AACGTGGTT element that was similar to AACGCGTT. Complexes 2 and 3 recognized a sequence in the left half of P2-89 ending in AACTTCC. This element was located entirely within functional element B. The overlap between the sites of promoter mutations and the approximate sites of complex formation suggests that the factors responsible for complex formation may also be important for promoter activity. Computer predictions suggest that element A might harbor sites for leucine zipper-type factors such as ATF1, CREB, and c-jun and others, while element B might harbor sites for ETS domain-containing factors such as c-ETS-1, Elk-1, Pu.1 and others. Although we have not yet verified these predictions, the identification of specific factors will be an important next step in understanding how this promoter is regulated.

### Rearrangement of the A and B elements and oocyte maturation

**Figure 7:**
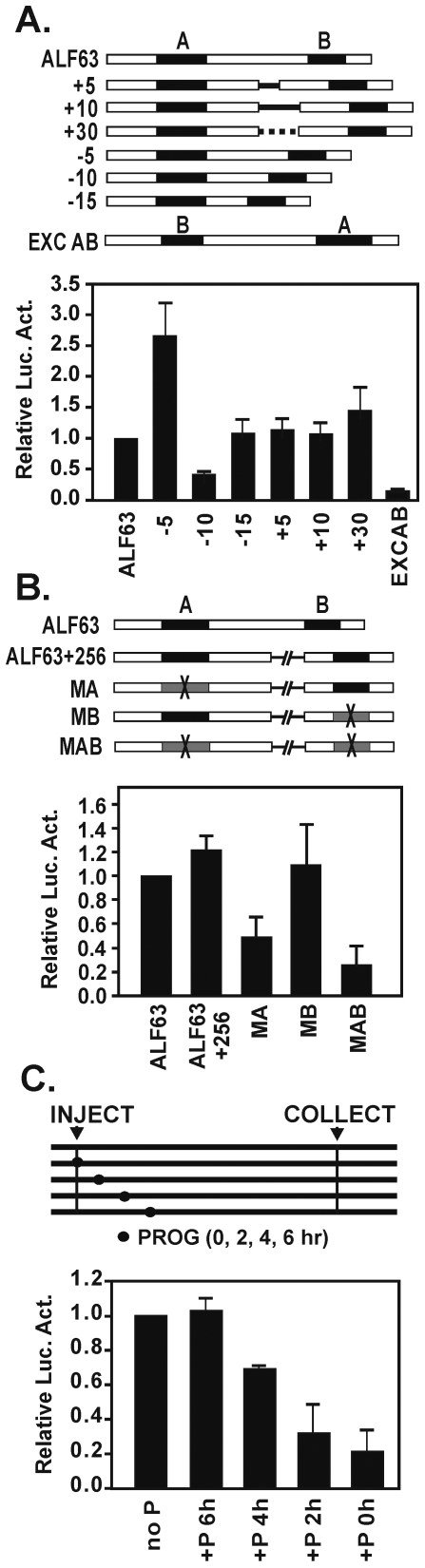
Effect of maturation and functional analysis of the A and B elements. (A) Deletion of sequences between the A and B elements and the introduction of sequences between the two elements resulted in increased activity for the −5 construct and lowered activity for the −10 construct. The remaining constructs (−15, +5, +10, and +15) showed activity similar to the control. An exchange of elements (EXCAB) led to loss of activity. (B) Separation of the A and B elements using a 256 bp insert resulted in an activity equivalent to the wild type control. Mutation of the repositioned A element (MA) and a combined AB mutant (MAB) resulted in the loss of promoter activity. (C) The effect of oocyte maturation on transcription activity of the ALF85 promoter. Progesterone (P) was added at 2 hour intervals relative to the time of microinjection.

We also examined the activity of constructs in which the relative locations of the A and B elements had been altered. Mutations in which the orientation was maintained but the spacing was altered showed a modest increase in activity for the −5 deletion, a slight decrease for the −10 deletion, and unaltered levels for the −15, +5, +10, and +30 constructs (Figure 7A). Separation of the A and B elements by insertion of a 256 spacer showed activity similar to wildtype, and individual mutations of the A element in the MA construct and in the MAB construct showed diminished activity (Figure 7B). The results show that the context of the A and B elements is important and that the rearrangements have complex effects on activity.

The activity of the ALF promoter was also examined in oocytes induced to mature by the addition of progesterone (Figure 7C). In particular, the farther cells were into the maturation program at the time of injection, the greater the reduction in promoter activity. This result is consistent with the idea that maturation initiates a period of transcriptional quiescence during which events are primarily driven by translational activation of a pool of stored maternal RNAs.

## Discussion

**Figure 8:**
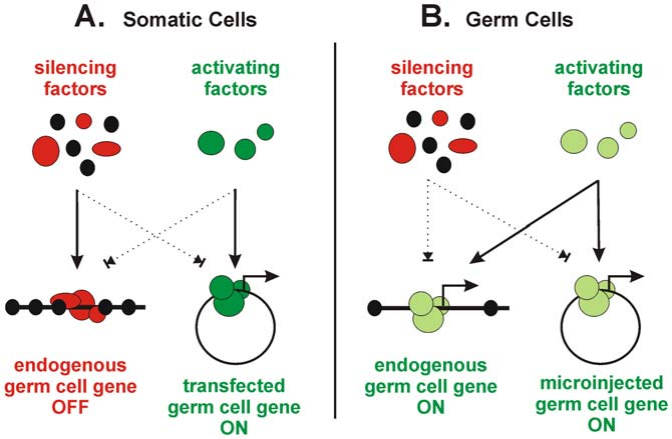
Effects of activating and repressing regulatory factors on endogenous and introduced promoter DNA in somatic cells and germ cells. Silencing factors are red/black while activating factors are green. Solid lines show paths of function while dashed lines indicate either absence or failure to function. (A) Silencing factors do not repress an introduced germ cell gene promoter in somatic cells, allowing activating factors to (inappropriately) drive transcription. (B) Silencing factors are absent or do not function on endogenous and introduced promoters in germ cells, while activating factors result in transcription from both types of DNA.

Studies on the mechanisms of transcription require a source of cells or cell extracts in which the promoter of interest displays the correct pattern of expression and inducibility. In the case of germ cell promoters this would ideally involve spermatocytes and oocytes. However, these cells undergo meiosis and cannot be propagated in cell culture. This has led to the use of cell-free extracts from whole testis, a tissue which contains germ cells in many stages of differentiation together with associated somatic cells, as well as the use of somatic cells which, although capable of transcribing transfected germ cell promoters, normally exhibit silencing of endogenous germ cell genes. Although candidate regulators can be been identified by such approaches, it has generally been difficult to prove that those factors are important for expression in the subpopulation of germ cells where the target gene is actually on. To begin to address these issues, we describe efforts to characterize a germ cell promoter by matching it to a cell type, in this case oocytes from *X. laevis*, where it is normally on. A summary of how somatic and germ cell transcription factors activate or silence endogenous and exogenous germ cell promoters is illustrated in Figure 8.

The use of *Xenopus* oocytes has a number of unique advantages. First, early studies of gene expression in these cells were based on the recognition that they are transcriptionally very active. Such studies helped define core promoter elements such as the TATA box and were important for developing methods of oocyte preparation and microinjection [10], [12]. Second, recent studies have shown that the basal transcription machinery present in oocytes is distinct from that in somatic cells [1]. This machinery includes a set of oocyte-specific germ cell substitutes for core promoter recognition factors like TBP, TFIIA, and TAFs [15], [17], [25]. These variants replace their somatic counterparts and are therefore critical for the recognition and regulation of germ cell genes. The presence of these variants in oocytes means that these cells provide an environment where it is possible to match a germ cell promoter to the set of factors (e.g. TRF3, ALF, etc.) that normally control its expression (Figure 8). Third, oocytes can be isolated in quantities sufficient for the preparation of cell-free extracts needed for *in vitro* protein-DNA interaction assays. This would not be as easily possible with mammalian oocytes because such cells must be individually dissected from ovarian tissue. Disadvantages to the system include the need for sufficient experimental replication of transcription assays to compensate for microinjection damage or frog-to-frog variation in oocyte quality. In addition, the *X. laevis* genome sequence is not available, and orthologous germ cell promoters from frogs and mammals, although conserved in terms of expression patterns, may diverge at the sequence level. On balance however, the ease of oocyte purification and the fact that they contain regulatory factors that are physiologically relevant make these cells an attractive system to address questions about the biochemistry of germ cell gene expression.

Interestingly, alignment of ALF promoter sequences from twelve diverse species showed poor similarity when all were included, and the similarity between two related frog species *X. laevis* and *X. troplicalis* was itself only about only 60%. The two regions of homology identified in the two frog species, as well as the CGCAAAA motif that appeared to line up with a conserved TATA-like TTCAAAA sequence in mammalian promoters, were not critical for transcription activity in oocytes. These observations raise questions about the role of conserved and nonconserved elements in mediating germ cell-specific expression. One possibility is that divergent regions of the promoter might be responsible for germ cell specific gene expression while conserved motifs might be important for somatic silencing. Germ cell-expressed genes and somatic cell-expressed genes evolve at different rates, and we speculate that the greater conservation of somatic factors would require a correspondingly greater conservation of target sites whereas more rapidly evolving germ cell factors might coevolve with rapidly changing regulatory sites. Since the current study was limited to oocytes, however, we do not yet know if the sequences active for expression are also responsible for somatic silencing, or if silencing might be due to a separate, more conserved region of the promoter.

Deletion analysis demonstrates that the minimal active region is only about 63 base pairs, about half that described for the mouse ALF gene using transgenic experiments [22]. The conclusions reinforce results of previous studies which have shown germ cell promoters to be quite small. Interestingly, transcription start site mapping experiments for the endogenous ALF gene suggest that initiation occurs at sites adjacent to or upstream of the active promoter domain defined in construct microinjection experiments. Further, bisulfite sequencing experiments to assay methylation status show that the endogenous promoter is relatively demethylated in DNA isolated from purified oocytes, consistent with the general correlation between demethylation and germ cell expression [24], [26], [27]. Overall, these similarities suggest that germ cell gene regulation in *Xenopus* oocytes is similar to other germ cell expression systems, including those of mammals.

Fine scale mutational analysis revealed two regions of the promoter (A and B) that, when altered, resulted in changes in both RNA levels and luciferase reporter activity. The results suggest that these two domains, despite their proximity to the 5′-UTR and ATG codon, are likely to be promoter regulatory elements rather than translational regulatory elements. Moreover, oligonucleotide competition assays showed that oocyte extracts contain factors that interact specifically with sequences within or adjacent to the A and B elements. Although computer analysis provides predictions about what these factors might be, these predictions will need to be verified in future studies using factor-specific antibodies and purification procedures. The promoter must also be recognized by oocyte-specific basal factor variants such as TRF3 and ALF in order to form a complete, transcriptionally active, preinitiation complex. Indeed, chromatin immunoprecipitation assays have demonstrated interactions between one such factor, TRF3, and a microinjected H2B promoter [17]. Whether the A and B elements are involved in direct interactions with the core transcription machinery or whether the complexes identified in bandshift assays mediate later steps in preinitiation complex assembly are interesting questions that can be addressed in future studies.

Previous efforts to characterize germ cell promoters have emphasized their relatively small size and sequence diversity and have identified many site-specific transcription factors possibly involved in their regulation. The current paper extends this work using an approach in which a germ cell promoter is introduced into a cell type, *Xenopus* oocytes, where the endogenous gene itself is naturally active. The results demonstrate the feasibility of the approach and define a very short *Xenopus*-specific germ cell promoter that can be used as a model to study regulatory factors and other mechanisms that are important for germ cell gene regulation.
